# Impact of NPM, TFF3 and TACC1 on the Prognosis of Patients with Primary Gastric Cancer

**DOI:** 10.1371/journal.pone.0082136

**Published:** 2013-12-16

**Authors:** Aiping Ding, Wenwen Zhao, Xiaoli Shi, Ruyong Yao, Fang Zhou, Lu Yue, Shihai Liu, Wensheng Qiu

**Affiliations:** 1 Department of Oncology, Affiliated Hospital of Qingdao University School of Medicine, Qingdao, People’s Republic of China; 2 Central Laboratory, Affiliated Hospital of Qingdao University School of Medicine, Qingdao, People’s Republic of China; 3 Qingdao Cancer Hospital, Shibei City, Qingdao, Shandong Province, China; INRS, Canada

## Abstract

**Background:**

NPM, TFF3 and TACC1 are molecular markers that play important roles in cell differentiation. Herein, we investigated their prognostic impact in patients with primary gastric cancer (GC) and determined whether they could be used as markers of more aggressive gastric carcinomas by detecting the extent of expression in human gastric carcinoma samples.

**Methodology/Principal Findings:**

Tumor tissue specimens from 142 GC patients were retrospectively retrieved and immunohistochemically evaluated. Correlations between NPM, TFF3 and TACC1 over-expression and clincopathologic parameters, and their prognostic values were investigated with χ^2^, Kaplan-Meier method, and Cox uni- and multivariate survival models. NPM, TFF3 and TACC1 expression was significantly higher in GC patients with poorly differentiated histologic type than that in patients with well differentiated histologic type. NPM expression was significantly higher in patients with hepatic metastasis or recurrence than that in patients without metastasis. TFF3 expression was significantly higher in patients with positive lymph node metastasis than that in patients with negative lymph node metastasis. Age, lymph node metastasis, and TFF3 and TACC1 over-expression were significantly correlated with low survival (P<0.05, P<0.05, P = 0.005 and P = 0.009, respectively). Multivariate analysis showed that lymph node metastasis and TFF3 and TACC1 over-expression were independent prognostic factors.

**Conclusions:**

TFF3 and TACC1 over-expression in epithelial cells of surgically resected GC tissues was an independent predictor of short survival in GC patients. The prognosis was poorer in patients with positive expression of both TFF3 and TACC1 than that in patients with positive expression of TFF3 or TACC1 alone, or with negative expression of TFF3 and TACC1.

## Introduction

Gastric cancer (GC) is the 4^th^ most common cancer worldwide (7.8% of all cancers) and the 2^nd^ leading cause of cancer death (9.7% of all cancer deaths) worldwide [1]. Although surgical resection is a viable option for early-stage GC patients, the control of GC progression remains difficult [2], [3]. The pathogenesis of GC is associated with multiple factors. Recently, various biological factors involved in the pathogenesis of GC have been identified, but their clinical relevance has not been confirmed. A better understanding of the biological basis of GC would be helpful.

Nucleophosmin (NMP), also known as numatrin or NO38, is a member of the nucleoplasmin (NPM) family. It is a nucleolar phosphoprotein constantly shuttling between the nucleolus and cytoplasm [4]. NPM exerts many functions, including generation of ribosomes, maintenance of genomic integrity, and transportation of proteins into the nucleus [5]. Therefore, the nucleophosmin/B23 gene (B23) seems to be involved in the control of cell growth, differentiation and programmed cell death [6], [7]. NMP is overexpressed or mutated in human cancer cells, and is therefore a candidate prognostic marker in colon, ovarian and prostate cancers [8], [9]. However, since most of these conclusions were prevalently based on indirect evidence with in vitro models, the exact contribution of NPM to tumorigenesis is far from clear largely due to a lack of appropriate clinical studies.

Trefoil factor 3 (TFF3) is a member of the TFF gene family, which encodes a series of small mucin-associated polypeptides [10]. TFF3 is mainly present in the gastrointestinal tract and other epithelial tissues, and is known to play an important role in maintaining mucosal integrity [11]. TFF3 is supposed to enhance cell migration through modulating functions of E-cadherin/catenin complexes [12]. Recently, TFFs have been reported to be overexpressed at both gene and protein levels in human neoplasms, including intestinal, pancreatic and prostate cancers.

Transforming acidic coiled*-*coil 1 (TACC1) was originally identified as the sole coding sequence consistently found within the 8p11 human breast cancer amplicon [13]. It is expressed at high levels during embryogenesis and then down-regulated in differentiated tissues [13]–[15]. TACC1 is involved in several cancers including breast and ovarian cancers and leukemia. In a recent study [16], TACC1 was found to be up-regulated and act as an oncogene in breast and ovarian cancers. However, a recent serial analysis of gene expression (SAGE) suggested that TACC1 was down-regulated in ovarian tumors and ovarian cancer cell lines [17]. Therefore, whether TCAA1 functions as an oncogene [16] or a tumor suppressor [18] is highly cancer type-dependent [13].

In the present study, we investigated the expression levels of NPM, TFF3 and TACC1 in GC patients, and also analyzed their potential correlations with clinical features and overall survival (OS) of GC patients.

## Materials and Methods

### Patients and Clinical Samples

Included in this study were 142 patients (93 male and 49 female) who received surgical resection of primary GC at the department of general surgery in our hospital between July 2007 and September 2009. The study protocol was approved by the Ethics Committee of the Affiliated Hospital of Qingdao University School of Medicine (Qingdao, China). Patients had signed an informed consent. The median age was 59 years (27 to 89 years). Tumor specimens were fixed in 10% formaldehyde solution and embedded in paraffin. None of the patients had received chemotherapy or radiotherapy before surgery. The pathologic diagnoses and classifications were made according to the Japanese Classification of Gastric Carcinoma. Complete demographic and clinical data were collected retrospectively. “Early cancer” was defined as cancer invasion limited to the submucosa, and “advanced cancer” was defined as cancer invasion into the muscularis propria or serosa. The end date of the follow-up was July 2012, and the median follow-up period for OS was 47 months (range 3–59 months) with a 3-year postsurgical survival rate of 64.2%.

### Immunohistochemistry

Array blocks were sliced into 4-μm sections, deparaffinized and hydrated in graded ethanol. Antigen retrieval was performed by immersing the slides in citrate buffer (Ph 6.0) and microwaving for 10 min. Deparaffinized and hydrated tissue sections were treated with 0.3% hydrogen peroxidase in methanol for 15 min, washed in PBS and incubated with the primary antibodies against NPM, TFF3 and TACC1 for overnight at 4°C (Table 1). Antibody staining was detected with the avidin-biotin-peroxidase complex (PV9005, mouse hypersensitivity, Beijing fir Jinqiao, China). Diaminobenzidine was used to visualize peroxidase deposition at the antigenic site, and these sections were counterstained with hematoxylin.

**Table 1:** Antibody used in this study.

Antigen	Antibody	Clone	Catalogue no	Source	Dilution
NPM	Anti-Nucleophosmin antibody	Mouse monoclonal	ab10530	abcam	1∶100
TFF3	Anti-Trefoil Factor 3 antibody	Mouse monoclonal	ab57752	abcam	1∶100
TACC1	TACC1 (E41) pAb	Rabbit	BS2644	abcam	1∶100

Immunohistochemical staining was assessed semi-quantitatively by measuring both the intensity of the staining (0, 1, 2, or 3) and the extent of staining (0, 0%; 1, 0–10%; 2, 10–50%; 3, 50–100%). The scores for the intensity and the extent of staining were multiplied to give a weighted score for each case (maximum possible, 9). For the statistical analysis, the weighted scores were grouped in two categories where scores of 0 to 3 were considered negative, and 4 to 9 positive.

### Statistical Analysis

All statistical analyses were performed using the statistical package SAS version 9.2. The X2 test and Fisher’s Exact test were used to examine correlations between the expression of the molecular markers and various clinicopathological parameters. Univariate analysis was performed by using the Kaplan–Meier method, and statistical significance between survival curves was assessed by the log rank test. OS was determined from the date of surgery to the time of cancer death. To assess the independent values of different variables on survival, multivariate analysis was carried out in the presence of other variables using the Cox proportional hazards model. Only variables of significant value from the univariate analysis were entered into the Cox regression analysis. p<0.05 was considered statistically significant.

## Results

### NPM, TFF3 and TACC1 Expression Patterns in Tumor Cells

**Figure 1:**
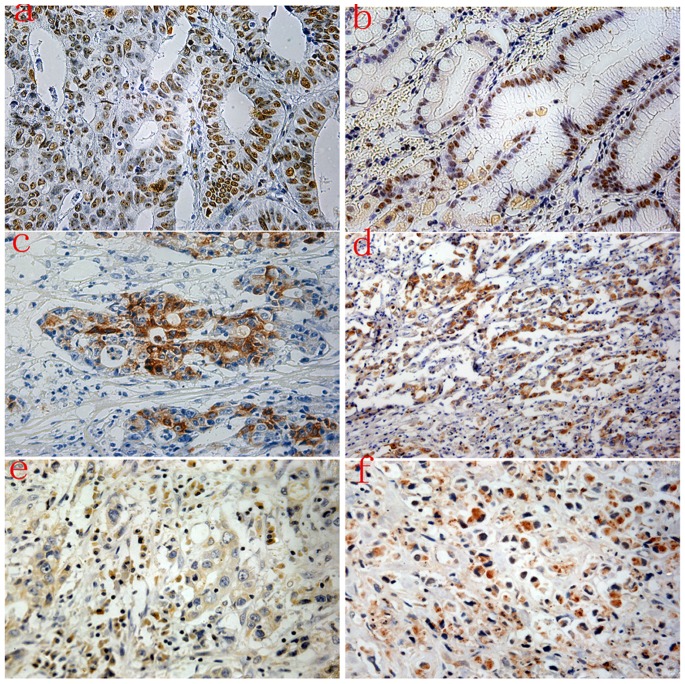
Immunohistochemical expression levels of NPM, TFF3 and TACC1 in gastric cancer. Strong staining of NPM was observed in the nucleolus, nucleus and cytoplasm of tumor epithelial cells (a, b). High expression of TFF3 (c and d) was predominantly observed in the cytoplasm of tumor epithelial cells. TACC1 was expressed in the cytoplasm of tumor epithelial cells (e and f).

Expression of NPM, TFF3 and TACC1 was measured by semi-quantitative IHC analyses. NPM was localized in the nucleolus, nucleus and cytoplasm of tumor epithelial cells (positive 47% vs. 53% negative) (Figure. 1). TFF3 was expressed in the cytoplasm of tumor epithelial cells (positive 44% vs. 56% negative), and no staining was observed in the nucleus (Figure. 1). TACC1 was expressed in the cytoplasm of tumor epithelial cells (positive 49% vs. 51% negative) (Figure. 1), and no staining was observed in the nucleus.

### Patient Clinicopathological Variables

The median age of the patients in this study was 59 years (27 to 89 years). The median follow-up overall survival was 47 months (range 3–59 months), and the 3-year postsurgical survival rate was 64.2%. The median tumor size was 35 mm (range 15–130 mm). With regard to tumor stage, 14% (20/142) of the patients were in early cancer and 86% (122/142) were in advanced cancer. Lymph node metastasis was observed in 55% (78/142) of the patients. Distant metastases, including hepatic metastasis, venous invasion and peritoneal metastasis, occurred in 15 patients.

### Correlations between Clinicopathological Data and the Expressions of NPM, TFF3 and TACC1

NPM, TFF3 and TACC1 expressions were significantly higher in patients with poor differentiated histologic type GC than those in patients with well differentiated histologic type GC (NPM, 0.88 vs. 0.12; TFF3, 0.90 vs. 0.10; TACC1, 0.90 vs. 0.10; P<0.05). Only NPM expression was significantly higher in patients with hepatic metastasis (0.88 vs. 0.12; P<0.05) or recurrence (0.73 vs. 0.27; P<0.05). TFF3 was significantly higher in patients with positive lymph node metastasis than that in patients with negative lymph node metastasis (TFF3, 0.61 vs. 0.39; p<0.05). TACC1 expression correlated significantly with venous invasion (0.88 vs. 0.12; P<0.05) and female patients (0.69 vs. 0.31; P<0.05). Clinical and histopathological variables are shown in Table 2.

**Table 2:** Correlation between clinicopathological factors and NPM, TFF3 and TACC1 expressions.

Clinicopathological factors	NPM		P-value	TFF3		P-value	TACC1		P-value
	Positive	Negative		Positive	Negative		Positive	Negative	
	N = 67	N = 75		N = 63	N = 79		N = 70	N = 72	
Age									
≤60	34	35	P>0.05	27	42	P>0.05	31	38	P>0.05
>60	33	40		36	37		39	34	
Gender									
Male	48	45	P>0.05	38	55	P>0.05	36	57	P<0.05
Female	19	30		25	24		34	15	
Tumor diameter									
≤50 mm (small)	47	45	P>0.05	39	53	P>0.05	45	47	P>0.05
>51 mm (large)	20	30		24	26		25	25	
Tumor depth									
Early cancer (T**1)	12	8	P>0.05	10	10	P>0.05	13	7	P>0.05
Advanced cancer (T2–T4)	55	67		53	69		57	65	
Histologic type									
Well moderately	8	19	P<0.05	6	21	P<0.05	7	20	P<0.05
Poor, others	59	56		57	58		63	52	
Peritoneal metastasis									
Negative	61	73	P>0.05	58	76	P>0.05	66	68	P>0.05
Positive	6	2		5	3		4	4	
Venous invasion									
Negative	65	69	P>0.05	60	74	P>0.05	63	71	P<0.05
Positive	2	6		3	5		7	1	
Lymph node metastasis									
Negative	26	38	P>0.05	22	42	P<0.05	29	35	P>0.05
Positive	41	37		41	37		41	37	
Hepatic metastasis									
Negative	60	74	P<0.05	59	75	P>0.05	64	70	P>0.05
Positive	7	1		4	4		6	2	
Recurrence									
Negative	56	71	P<0.05	55	72	P>0.05	61	66	P>0.05
Positive	11	4		8	7		9	6	

P<0.05 Statistically significant;

T: tumor size.

### Univariate Analysis

**Figure 2:**
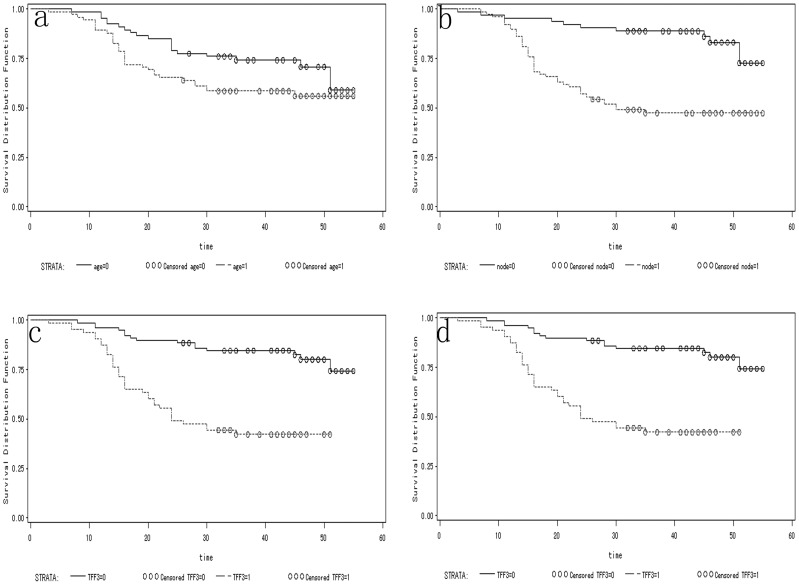
Kaplan-Meier overall survival curves of 142 GC patients according to age (a), lymph node metastasis (b), TFF3 expression (c), and TACC1 expression (d) (P<0.05). Abbreviations: age = 0 means age≤60 years; age = 1 means age>60 years. node = 0 means no lymph node metastasis; node = 1 means lymph node metastasis.

Univariate analyses showed that age, lymph node metastasis, TFF3 over-expression and TACC1 over-expression were significantly correlated with a short survival time (Table 3, Figure 2). For statistical analysis, the designated “high-risk group” had an altered expression of 2 or 3 proteins. the designated “low-risk group” had an altered expression of 0 or 1 proteins. The prognosis was poorer in patients with positive co-expression of NPM/TFF3, TFF3/TACC1, NPM/TACC1 and NPM/TFF3/TACC1 than that in patients with positive expression of NPM, TFF3 or TACC1 alone, or with negative co-expression of these markers (Table 3).

**Table 3:** Univariate analysis with respect to overall survival in 142(univariate analysis; log-rank test).

Characteristics			Patients (n)	Patients (%)	p-value
Age (years)					P<0.05
	≤60		69	49	
	>60		73	51	
Gender					P>0.05
	Male		93	65	
	Female		49	35	
Histology					P>0.05
	Well moderately		27	19	
	Poor, others		115	81	
Tumor diameter					P>0.05
	≤50 mm (small)		92	65	
	>51 mm (large)		50	35	
Stage					P>0.05
	Early cancer (T**1)		20	14	
	Advanced cancer (T2–T4)		122	86	
Peritoneal metastasis					P<0.05
	Negative		134	94	
	Positive		8	6	
Venous invasion					P>0.05
	Negative		134	94	
	Positive		8	6	
Lymph node metastasis					P<0.05
	Negative		64	45	
	Positive		78	55	
Hepatic metastasis					P<0.05
	Negative		134	94	
	Positive		8	6	
Recurrence					P<0.05
	Negative		127	89	
	Positive		15	11	
NPM					P>0.05
	Negative		75	53	
	Positive		67	47	
TFF3					P<0.001
	Negative		79	56	
	Positive		63	44	
TACC1					P<0.001
	Negative		72	51	
	Positive		70	49	
NPM/TFF3					P<0.05
	Co-negative		47	33	
	Single-positive		60	42	
	Co-positive		35	25	
TFF3/TACC1					P<0.001
	Co-negative		54	38	
	Single-positive		43	30	
	Co-positive		45	32	
NPM/TACC1					P<0.05
	Co-negative		47	33	
	Single-positive		54	38	
	Co-positive		41	29	
NPM/TFF3/TACC1					P<0.001
	Co-negative		39	27	
	Single-positive		79	56	
	Co-positive		24	17	

### Multivariate Analysis

Multivariate analysis showed that lymph node metastasis and the expression of TFF3 and TACC1 in tumor epithelial cells were independent prognostic factors for overall survival (Table 4), and that the hazard ratio (HR) of tumor-related death increased by 3.409 fold in FF3 group (P<0.001; 95% CI 2.387–8.143). The 3.311-fold increase in HR of tumor-related death in lymph node metastasis group was obviouslyly significant (P<0.001; 95% CI 2.148–8.651) (Table 4). The 2.278-fold increase in HR of tumor-related death in TACC1 group was also obviously significant (P<0.001; 95% CI 1.803–5.959).

**Table 4:** Multivariate analysis of overall survival in 142(multivariate analysis; log-rank test).

Factors	Hazardratio	95% confidenceinterval (CI)	p-value
Lymph node metastasis	4.311	2.148–8.651	<0.001
TFF3	4.409	2.387–8.143	<0.001
TACC1	3.278	1.803–5.959	<0.001

= 0 means TFF3-negative expression; TFF3 = 1 means TFF3-positive expression. TACC1 = 0 means TACC1-negative expression; TACC1 = 1 means TACC1-positive expression. P-values were calculated by the log-rank test. TFF3

## Discussion

Accordingly, examination of expression of specific genes in gastric carcinoma cells could help you understand the histopathological features of GC.Consequently, it is essential to study the different molecular markers to be able to understand the mechanism of tumorigenesis and metastases. Therefore, we sought out to test a panel of three molecular markers associated with tumorigenesis.

Nucleophosmin/B23 expression is a nucleolar phosphoprotein constantly shuttling between the nucleolus and cytoplasm [4], and at the RNA and protein levels may contribute to the onset of cancer [19], [20], NPM was previously suggested as a prognostic factor for poor survival and is related to tumor progression and resistance [21], [22]. However, the results obtained in the present study differed in several important respects from these earlier studies. We found a worse outcome in patients with NPM over-expression, but it is not an independent prognosis marker, possibly because of the biological variation of protein expression in different areas of tumor tissue. In our study, NPM over-expression was observed in 73% patients with recurrence, indicating that NPM over-expression was significantly associated with recurrence. The study of Tsui et al [23] also showed that high NPM/B23 level was correlated with recurrence of bladder cancer. In addition, it is intriguing to speculate that NPM might was correlated with promote hepatic metastasis of this cancer. The result of the present study indicates that NPM over-expression may prove to be a predictor for bad prognosis of GC, although the exact underlying mechanism remains unclear.

Previous studies [24], [25] showed that TFF3 was frequently over-expressed in breast cancer and other primary tumors. Our results showed that 44% of gastric carcinomas expressed TFF3, and more importantly that TFF3 over-expression in GC was an independent negative prognostic indicator for OS. The risk of death was significantly increased in patients with TFF3 over-expression. In addition, TFF3 over-expression was the second highest risk next to lymph node metastasis for postoperative survival. Significant correlations between TFF3 over-expression and poor prognosis have been previously reported in cholangiocarcinoma [26] and endometrioid endometrial carcinoma [27], which is consistent with our result. Our data also suggest that TFF3 over-expression was correlated with more aggressive clinicopathological parameters. For example, TFF3 over-expression was detected in approximately 50% of the patients with positive lymph node metastasis *vs.* one-third in patients with negative lymph node metastasis, indicating that TFF3 over-expression plays an important role in lymph node metastasis in GC patients. Further studies on molecular mechanism are needed to define more precisely the role of trefoil peptide expression in tumor progression and metastasis.

Previous studies [28], [29] have suggested the possible effect of TACC1 on cell proliferation and its carcinogenetic properties. However, to the best of our knowledge, there are no other studies reporting the correlation between TACC1 over-expression and clinical significance in GC patients. One of the significant findings of this study is the great gender-related impact on TACC1 over-expression, which shows that 69% of the female GC patients exhibited positive TACC1 expression. In addition, the number of female patients with TACC1 over-expression was twice that in male patients with negative TACC1 expression. However, TACC1 over-expression had no significant impact on the survival with respect to the gender as opposed to the significant impact on the survival of different age groups, suggesting that there may be other mechanisms affecting the survival of GC patients. The result of our multivariate analysis showed that TACC1 over-expression was an independent prognostic factor of GC, which is consistent with the earlier finding on the prognostic significance of TACC1 observed in ovarian tumors [30]. In addition, our data provide the evidence that TACC1 over-expression is associated with venous invasion, implying its possible role in tumor metastasis.

Approximately half of cancer specimens were found with co-expression of these two markers. Univariate analysis shows that the high-risk group was significantly associated with shorter patient overall survival, and that high-risk group exhibits a poorer prognosis than low-risk group. Whether the combined expression of NPM, TFF3 and TACC1 contributes a growth advantage of tumour must be further determined by in vitro and clinical studies.

In conclusion, TFF3 and TACC1 over-expression in tumor epithelial cells of surgically resected gastic adenocarcinoma could independently predict a shorter survival. NPM over-expression is significantly correlated with the clinical pathology features of GC. These observations indicate that over-expression is crucial for the pathogenesis and development of GC. Additional investigations are needed to provide a better foundation for the development of more effective therapeutic strategies for GC and other human malignancies.
